# Maternal hypothyroidism during pregnancy alters the function of the retinol-binding protein 4-mediated mitochondrial permeability conversion pore in the kidneys of offspring rats

**DOI:** 10.6061/clinics/2021/e2096

**Published:** 2021-01-18

**Authors:** Danyan Chen, Li Li, Fang Ren, Rongxi Huang, Hua Gan, Huacong Deng, Hongman Wang

**Affiliations:** I Departments of Endocrinology, Chongqing General Hospital, University of Chinese Academy of Sciences, Departments of EndocrinologyChongqing General HospitalUniversity of Chinese Academy of SciencesChinaChina; II Departments of Emergency, Chongqing General Hospital, University of Chinese Academy of Sciences, Departments of EmergencyChongqing General HospitalUniversity of Chinese Academy of SciencesChinaChina.; III Departments of Nephrology, the First Affiliated Hospital of Chongqing Medical University, Departments of Nephrologythe First Affiliated Hospital of Chongqing Medical UniversityChinaChina; IV Departments of Endocrinology, the First Affiliated Hospital of Chongqing Medical University, Departments of Endocrinologythe First Affiliated Hospital of Chongqing Medical UniversityChinaChina

**Keywords:** Maternal Hypothyroidism, Pregnancy, Offspring, mPTP, RBP4/PiC/SIRT3 Pathway

## Abstract

**OBJECTIVES:**

To determine the role of the RBP4/PiC/SIRT3 signaling pathway in the opening of the mitochondria permeability transition pore (mPTP) in offspring rats with hypothyroidism during pregnancy.

**METHODS:**

Sixty Sprague-Dawley (SD) rats were employed in this study. Pregnancy was deemed successful when a sperm was found in the uterus. After one week of pregnancy, offspring rats were divided into the following groups: overall hypothyroidism group (OH group), subclinical hypothyroidism group (SCH group), and normal control group (CON group). The establishment of the hypothyroidism model was confirmed when the serum thyroid stimulating hormone (TSH) levels were higher than normal value and TT4 level was within the normal range. The renal mitochondria of offspring rats were extracted on the 14^th^ postnatal day (P14) and 35^th^ postnatal day (P35).

**RESULTS:**

At P14, no significant differences in the degree of mPTP opening and expression of phosphoric acid carrier vector (PiC) were detected between the rats in the OH group and the SCH group. However, the expression level of silent mating-type information regulation 3 homolog (SIRT3) was markedly reduced. Retinol-binding protein 4 (RBP4) expression increased in the rats from the OH group, relative to that in those from the SCH group. At P35, the degree of mPTP opening and the expression levels of PiC and RBP4 in the OH group were higher than those in the SCH group. However, SIRT3 expression in the OH group was lower than that observed in the SCH group.

**CONCLUSION:**

RBP4 plays an important role in early renal mitochondrial damage and renal impairment in rats suffering from hypothyroidism during pregnancy. The RBP4/PiC/SIRT3 pathway is thus involved in the opening of the renal mPTP in offspring rats with hyperthyroidism.

## INTRODUCTION

Owing to improvements in the economy and the popularization of iodized salt, the prevalence rate of thyroid disease has reduced considerably in the recent past(1). However, thyroid disease remains the second most prevalent endocrine disease in China (2). According to an epidemiological survey conducted by the Chinese Society of Endocrinology of the Chinese Medical Association, the overall prevalence rate of thyroid disease in Beijing, Shanghai, Guangzhou, and seven other cities was approximately 20% in 2010. Further, the prevalence rate of hypothyroidism increased from 3.8% (2006) to 6.5% (2010). To date, approximately 0.3-0.5% of pregnant women suffer from hypothyroidism while 2-5% of pregnant women suffer from subclinical hypothyroidism (3,4). The domestic prevalence rate of hypothyroidism during pregnancy is approximately 1%, while that of subclinical hypothyroidism exceeds 4% (5). During the early stage of pregnancy (12-16 weeks), the thyroid of the mother is the only source of thyroid hormones for fetal growth. As a result, mild thyroid hormone deficiency may lead to irreversible damages to neural development, learning, memory, and cognition in offspring (6-8).

Thyroid hormones have been suggested to affect kidney development and the balance of water and electrolytes. Under normal physiological conditions, most thyroid hormones are combined with plasma proteins. However, the levels of the thyroid hormone are found to decrease in patients with thyroid disease. Multiple adverse reactions also occur simultaneously, including Na^+^-K^+^ pump synthetic activity reduction, increased peripheral vascular resistance, and organ/tissue ischemia. In the kidney, thyroid disease could result in multiple adverse reactions, such as renal blood flow reduction, glomerular basement membrane thickening, glomerular filtration rate reduction, and damage to the renal structure and functions (9). These adverse events are clinically manifested as abnormal renal function or proteinuria, ultimately affecting the Na^+^/Ca^2+^transporter functions of the renal cell membrane (10). As a result, they can induce electrolyte metabolic disturbances (11,12). Thyroid dysfunction could result in a decrease in the mitochondrial membrane and cause mitochondrial damage and fragmentation of the cristae and concentrated nucleus. Therefore, thyroid disease can potentially induce mitochondrial dysfunction and aggravate damage to intrinsic renal cells (13,14). The kidney is responsible for the metabolism of the thyroid hormone. When the renal structure and functions change, the thyroid hormone can be excreted with urine, inducing hypothyroidism. During the process of the above pathophysiology, cells can trigger the autophagy program, which can selectively degrade damaged or unnecessary mitochondria. Once the balance between the quantity and quality of the mitochondria is maintained, normal metabolic functions of the thyroid can occur.

Retinol-binding protein (RBP) is a new adipocyte factor that is composed of 181 amino acid residues. RBP belongs to the retinol-conjugated protein secreted in the retinol-binding protein family. Retinol-binding protein 4 (RBP4), which is synthesized by the liver and released into the blood, has been identified to be related to the development of metabolic syndrome, atherosclerosis, and hypothyroidism, owing to its interaction with retinol, thyroxine transporter, and cell surface receptors (15,16). However, only few studies have investigated its role in the opening/closing of the renal mitochondrion permeability transition pore (mPTP) and autophagy functions in rats suffering from hypothyroidism during pregnancy. Therefore, we opted to use a rat model to determine the role of RBP4 in mPTP opening in offspring rats that suffered from hypothyroidism during pregnancy.

## MATERIAL AND METHODS

### Animal experimental design

All experimental procedures were conducted in accordance with the Chinese legislation and the US National Institutes of Health guidelines for the use and care of experimental animals. All animal experiments were approved by the institutional ethical committee of Chongqing Academy of Medical Science. A total of 60 Sprague-Dawley (SD) rats (Female:Male=2:1, weight=200±20 g, Experimental Animal Center of Chongqing Academy of Medical Science, Chongqing) were employed in this study. All experimental protocols were reviewed and approved by the Chongqing General Hospital ethics committee. Female and male rats were mated and fed in an environment with a temperature of 21±2°C, a relative humidity of 50%, and a 12h-12h light-dark cycle (SPF Grade Animal Feeding Center of Laboratory Animal Department, Chongqing Medical University). The vaginal smears of offspring rats were observed microscopically after mating. Pregnancy was deemed successful when a sperm was found in the uterus. After one week of pregnancy, offspring rats were randomly divided into three groups: subclinical hypothyroidism group (SCH group), overall hypothyroidism group (OH group), and control group (CON group). In the OH group, the thyroid was completely removed, and blood was collected from the postorbital venous plexus at day 30 post-op. The successful establishment of the model was confirmed by a higher level of thyroid stimulating hormone (TSH) relative to that in the control group and a TT4 level <1.0 µg/dl (lower limit value of detection). In the SCH group, based on the successful development of the hypothyroidism rat model, 1.0 µg/100 g L-T4 (Sigma Inc., USA) was subcutaneously administered to each rat every day (17). The model was confirmed to be successfully established when the level of serum TSH was higher than the normal value to control group and the TT4 level was within the normal range. In the CON group, the thyroid was exposed during the operation. Rats were only fed 4-6 h after the operation. Additionally, 1.5% calcium gluconate was added to their drinking water to prevent low calcium seizures caused by the removal of the parathyroid gland. Delivery day was considered as P0. Blood was collected from the posterior orbital venous plexus of the offspring rats at P1. The levels of TT4 and TSH were measured throughout pregnancy. offspring rats in each group were weighed on the 14^th^ postnatal day (P14) and 35^th^ postnatal day (P35), and the serum TT4 and TSH levels were measured. Thereafter, decollation was performed on ice and the kidney tissue was retrieved for weighing. The following equation was used to calculate the kidney index: kidney index=kidney weight/body weight×1000. SD rats that mated three times without vaginal plug secretion were considered infertile.

### Mitochondria extraction

The kidney tissue was cut to form a minced sample and was subsequently suspended in a mitochondrial separation reagent containing 0.25 mg/mL pancreatin. The kidney tissues were mixed evenly and liquid albumin was added until a concentration of 10 mg/ml was achieved after 20 min of incubation on ice. The samples were then centrifuged at 1000×g for 30 s at 4°C. The supernatant was removed, and the mitochondrial separation reagent was added to flush the tissue fragments. A Dounee homogenizer (Beyotime Institute of Biotechnology, Shanghai, China) was used for homogenization (15 rounds of homogenization using a loose-type shaker followed by 10 rounds of homogenization using a compact-type shaker). The sample was centrifuged at 10000×g for 5 min at 4°C. The supernatant was transferred to another centrifuge tube and re-centrifuged at 10000×g for 10 min at 4°C. When the supernatant was removed, mitochondria remained as the sediment, which were stored at -70°C until further use.

### Assessment of the degree of mPTP opening degree by flow cytometry

The degree of mPTP opening was measured using a JC-1 Assay kit (Beyotime Institute of Biotechnology, Shanghai, China) according to the manufacturer's protocol. Immediately following ultrasonic treatment, cells were treated with 10 mg/mL JC-1 for 20 min at 37°C in dark. Fluorescence intensity was measured with a multimode microplate reader at excitation and emission wavelengths of 525 nm and 590 nm, respectively, for red fluorescence and 490 nm and 530 nm, respectively, for green fluorescence. Imaging was performed with a fluorescence microscope (Leica DM4 B&DM6 B, Germany).

### Real-Time Quantitative Polymerase Chain Reaction (RT-qPCR)

Total ribonucleic acid (RNA) was extracted with TRIzol reagent (TaKaRa Bio Inc., Kyoto, Japan), according to the manufacturer’s instructions. For RT-qPCR, deoxyribonucleic acid (cDNA) was synthesized using the Primescript RT-PCR kit (TaKaRa Bio Inc., Kyoto, Japan). The following primers (Shanghai Biotechnology Company, Shanghai, China) were used to detect rat phosphoric acid carrier vector (PiC), AMPK, and β-actin (internal control): PiC forward 5′-GTGGTTTGG-CTAAAGGATGG-3′ and reverse 5′-GGGCAATGTCAGCGAAGAA-3′; and β-actin forward 5′-CGTAAAGACCTCTATGCCAACA-3′ and reverse 5′-AGCCACCAATCCACA-CAGAG-3′. cDNAs were amplified using the following thermal cycle conditions: denaturation at 95°C for 1 min followed by 47 cycles of denaturation at 95°C for 10 s, annealing at 58°C for 10 s, polymerization at 72°C for 20 s, and a brief detection at 72°C. The signal was calculated based on the expression of β-actin (relative ratio=average copy number of the target gene in the sample/average copy number of β-actin).

### Protein expression of PiC, silent mating-type information regulation 3 homolog (SIRT3), and RBP4

Cellular proteins in three distinctive groups were extracted with 1% PMSF and RIPA lysis buffer (50 mM Tris-HCl [pH7.4], 150 mM NaCl, 1% NP-40, 0.1% SDS). Proteins were separated via sodium dodecyl sulfate-polyacrylamide gel electrophoresis (SDS-PAGE) on 10% gradient gels and subsequently transferred onto polyvinylidene difluoride membranes. The following antibodies were used: 1:500 dilution of the PiC antibody (Abnova, USA), 1:500 dilution of the SIRT3 antibody (CST, USA), 1:500 dilution of the RBP4 antibody (Abcam, USA), and 1:1000 dilution of the β-actin antibody (CST, USA). After incubation, the membranes were washed twice with washing buffer (phosphate-buffered saline and 0.05% Tween 20) for 15 min and incubated with a secondary antibody to horseradish peroxidase for 2 h at 25°C. The membranes were then washed three times with washing buffer for 15 min. Immunoreactivity was examined using an enhanced chemiluminescence (ECL) detection kit (PromoCell, Germany), according to the manufacturer’s instructions.

### Statistical Analysis

All data were analyzed using SPSS v23.0 software (SPSS, Chicago, IL, USA). Values are expressed as the mean±standard deviation. The enumeration data were analyzed using the chi-square test method. The difference among groups was determined using a t-test. A *p* value <0.05 was considered to indicate statistical significance.

## RESULTS

### Phenotypic and behavioral characteristics of the offspring rats

As shown in Table 1, the rats in the CON group showed good activity, fast weight gain, and normal hair development at P14 compared to those in the other groups at the same development time point. Significant abnormalities in activities and hair development were not detected in the rats at P14 in the SCH group compared to those in the CON group. However, rats lost weight (t=16.104, *p*<0.001) and the increased kidney indexes (t=5.025, *p*=0.005) were significantly different between the two groups. At P14, rats in the OH group were small, moved slowly, exhibited gait disturbances, and had a short tail. Further, their hairs developed slowly and were sparse. They were also recognized to have a lower than the rats in the SCH/CON group weight. However, the kidney indexes were higher in the SCH group than in the SCH group (weight: t=33.706, *p*<0.001; kidney indexes: t=5.139, *p*=0.005). Rats in the OH group were small, and their weight gain progressed slowly. In fact, their weight gain was significantly lower than those of rats of the same age in the CON group and SCH group at P35 (CON: t=51.779, *p*<0.001; SCH: t=53.872, *p*<0.001). However, the kidney indexes of rats in the OH group were significantly higher than those of rats of the same age in the CON group and SCH group (CON: t=9.324, *p*=0.002; SCH: t=7.764, *p*=0.003). The weights of rats of the same age in the CON group were compared to those of rats in the SCH group at P35; however, no statistical difference was found between the groups (weight: t=1.987, *p*=0.051; kidney indexes: t=2.115, *p*=0.035). The kidney indexes at P14 were significantly lower than those at P35 at different time points of development (OH: t=3.655, *p*=0.007; SCH: t=5.225, *p*<0.001; CON: t=10.127, *p*<0.001).

### TT4 and TSH levels in the rats

No statistically significant differences in serum TT4 (P14: t=1.107, *p*=0.075; P35: t=1.677, *p*=0.063) and TSH (P14: t=2.001, *p*=0.048; P35: t=1.779, *p*=0.059) levels were found between the CON group and the SCH group at P14 and P35. Moreover, serum TT4 level was decreased while TSH was increased in the OH group at P35 (TT4: t=11.900, *p*<0.001; TSH: t=19.677, *p*<0.001). Serum TT4 levels in the rats were also significantly higher at P14 than at P35 (OH: t=3.387, *p*=0.008; SCH: t=8.047, *p*<0.001; CON: t=6.141, *p*<0.001) while TSH level was significantly lower at P14 than that at P35 (OH: t=20.068, *p*<0.001; SCH: t=8.470, *p*<0.001; CON: t=4.104, *p*<0.001).

### Degree of mPTP opening (mitochondria swelling) and PiC mRNA expression

**Figure 1:**
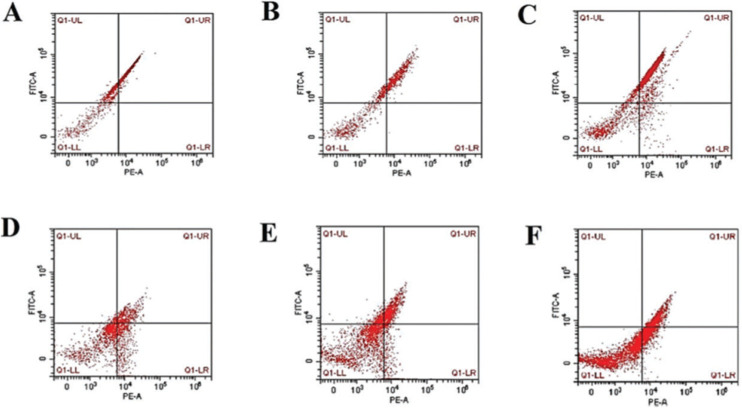
The degree of mitochondria swelling caused by mPTP opening in the different groups. Representative graphs from flow cytometry analysis demonstrating the degree of mitochondria swelling induced by mPTP opening in P14 and P35 of the CON group (A and D, respectively), SCH group (B and E, respectively), and OH group (C and F, respectively). Mitochondria permeability transition pore (mPTP); Control group (CON); Overall hypothyroidism group (OH); Subclinical hypothyroidism group (SCH); the 14th postnatal day (P14) ; the 35th postnatal day (P35).

**Figure 2:**
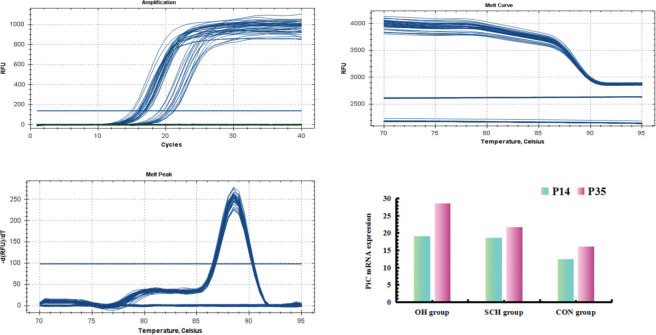
Comparison of mRNA expression of PiC in offspring rats in the OH group and SCH group. Phosphoric acid carrier vector (PiC); Overall hypothyroidism group (OH); Subclinical hypothyroidism group (SCH).

As shown in Table 2 and Figs. 1-2, higher levels of mPTP opening degree and PiC mRNA expression could be detected in the rats in the hypothyroidism group than in the CON group (*p*<0.05). At P35, mPTP opening degree and PiC mRNA expression in the OH group were respectively increased by 0.41-fold and 0.32-fold relative to those in the SCH group (mPTP opening degree: t=15.691, *p*<0.001; PiC mRNA expression: t=21.831, *p*<0.001). However, the mPTP opening degree and PiC mRNA expression levels in the rats at P14 were significantly lower than those at P35 (mPTP opening degree: OH: t=23.022, *p*<0.001; SCH: t=13.842, *p*<0.001; CON: t=3.403, *p*<0.01; PiC: OH: t=32.189, *p*<0.001; SCH: t=10.729, *p*<0.001; CON: t=10.971, *p*<0.001).

### PiC, SIRT3, and RBP4 protein expression

**Figure 3:**
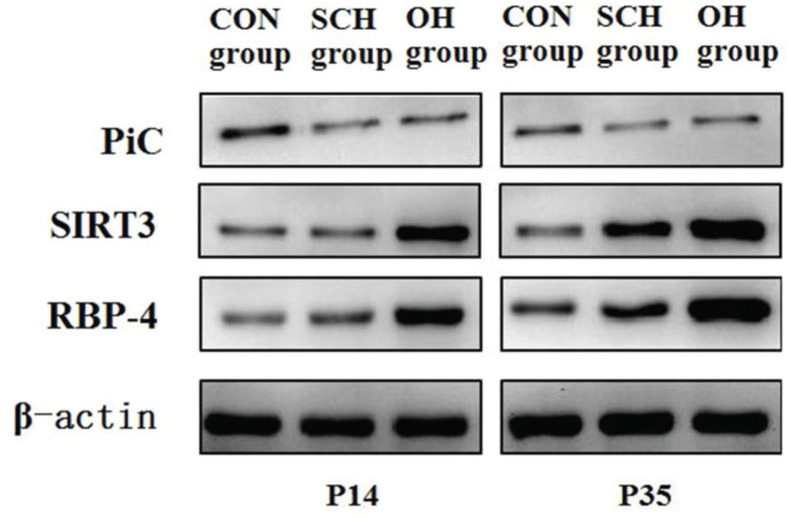
Protein expression levels of RBP4, PiC, and SIRT3 in the different groups. Retinol-binding protein 4 (RBP4); Phosphoric acid carrier vector (PiC); Silent mating-type information regulation 3 homolog (SIRT3).

As shown in Table 3 and Fig. 3, the expression levels of PiC and RBP4 in the rats in the CON group were significantly lower than those in the rats from the hypothyroidism groups at P14 and P35. However, SIRT3 expression in the CON group was significantly higher than that in the hypothyroidism group. Compared to the SCH group, the expression levels of SIRT3 and RBP4 in OH group decreased by 25% and increased by 37%, respectively (SIRT3: t=7.651, *p*<0.001; RBP4: t=14.277, *p*<0.001). At P35, there were significant differences in PiC, SIRT3, and RBP4 expression between the OH group and the SCH group (PiC: t=15.826, *p*<0.001; SIRT3: t=13.751, *p*<0.001; RBP4: t=24.282, *p*<0.001). Moreover, the expression levels of PiC and RBP4 were significantly lower than those at P35 (PiC: OH: t=24.836, *p*<0.001; SCH: t=18.203, *p*<0.001; CON: t=9.344, *p*<0.001; RBP4: OH: t=30.335, *p*<0.001; SCH: t=16.621, *p*<0.001; CON: t=9.161, *p*<0.001) among the groups at different time points. However, the expression level of SIRT3 was significantly higher than that at P35 (OH: t=13.868, *p*<0.001; SCH: t=9.107, *p*<0.001; CON: t=6.007, *p*<0.001).

## DISCUSSION

In the present study, we sought to investigate the effect of the RBP4/PiC/SIRT3 signaling pathway on mPTP opening in rats that suffered from hypothyroidism during pregnancy. Briefly, we first sought to successfully establish the overall hypothyroidism model in rats. Based on our results, RBP4 plays an important role in early renal mitochondrial damage and renal impairment in rats with hypothyroidism during pregnancy. Moreover, the RBP4/PiC/SIRT3 pathway was identified to be involved in the function of the renal mPTP of offspring rats with hypothyroidism during pregnancy.

In the field of pregnancy endocrinology, the influence of maternal hypothyroidism during pregnancy on the offspring has not been fully revealed. Previous studies have demonstrated that pregnancy has a significant effect on maternal thyroid function and volume. For example, the stimulation of human chorionic gonadotropin (HCG) on TSH receptors during pregnancy caused an estimated 50% increase in the demand for T4 and T3. Furthermore, the thyroid volume in the pregnancy period increased by 10%-40% and the demand for the thyroid hormone and daily iodine increased by approximately 50% (18,19). As a result, hypothyroidism develops in women with limited thyroid hormone during pregnancy. For patients who were diagnosed with hypothyroidism or subclinical hypothyroidism before pregnancy, issues with their thyroid hormone levels were inevitable. Mohebbati R et al. (20-22) suggested that in patients with hypothyroidism or subclinical hypothyroidism during pregnancy, the level of this hormone can return to normal through replacement therapy with levothyroxine. However, the effects of the altered functions of renal mitochondria in their offspring remain unclear. 

The mitochondria serve as the primary location of intracellular energy metabolism for cells with a double membrane and thus play a crucial role in cell differentiation, cell information transmission, cell apoptosis, cell growth, and cell cycle. Because mitochondrial DNA is easier to mutate than nuclear DNA, a low level of autophagy occurs in cells. Removing redundant or damaged mitochondria is essential for normal cell growth (23,24). Mitochondria autophagy is a selective macroautophagy that serves as a highly conserved self-digestion and metabolic process (25,26). The mPTP is a polyprotein composite structure that acts as a non-specific high-conductivity channel that stores Ca^2+^ between the inner and outer membranes of the mitochondria. The components of mPTP include the adenine nucleotide transporter on the mitochondrial inner membrane, voltage-dependent anion channel on the mitochondrial outer membrane, and cyclophilin D (Cyp D) in the mitochondrial matrix. Varanyuwatana P and Korotkov SM et al. indicated that Ca^2+^ and CypD can trigger changes in the mitochondrial PiC conformation, which induces mPTP opening via an interaction with the phosphoric acid transporter (27,28). When there is a lack of PiC, Pi fails to promote Ca^2+^ aggregation by the mitochondria. PiC might thus play an essential role in mPTP and Ca-Pi compound accumulation.

There are three open states of mPTP: 1) the fully closed state, this is when the mitochondrial membrane potential is completely closed; 2) a lower level of reversible open state that conducts an electric signal and a calcium signal; and 3) a high level of irreversible open state that causes irreversible changes in cells, even apoptosis. Under normal conditions, mPTP is in the fully closed state, which regulates energy metabolism by controlling the oxidative phosphorylation of cells, adjusts the permeability of the mitochondrial membrane, and maintains a relatively constant environment between the inner and outer ions. When the body is in a pathological state (hypothyroidism), Ca^2+^ overload and oxidative stress can result in a high concentration of Ca^2+^ in the mitochondrial matrix, which facilitates the combination of Cyp D and the adenylate transporter. Therefore, mPTP exists in the reversible open state (29). Our study findings suggest that there is no difference in the degree of mPTP opening and PiC mRNA expression between the OH group and SCH group at P14. However, a high level of mPTP was present in the open state at P35. Further, the degree of mPTP opening and PIC mRNA expression in the OH group were significantly higher than those in the SCH group. Mitochondrial membrane potential and osmotic equilibrium damage result in multiple adverse events, including mitochondrial matrix swelling, mitochondrial outer membrane rupture, disappearance of the mitochondrial cristae, and apoptosis-promoting cytokine release. Therefore, we speculate that the inhibition of mPTP opening can protect mitochondrial function and reduce renal damage in rats with hypothyroidism during pregnancy.

Recent studies have indicated that RBP4 is synthesized by the liver. In fact, RBP4 can be filtered freely by the glomerulus and reabsorbed by the proximal convoluted tubule. Previously, the levels of RBP4 in the serum and urine of patients with hypothyroidism were found to increase (15), which ultimately aligns with our study results. In the present study, the expression level of RBP4 was significantly higher in the OH group than the SCH group at P14 and P35. However, its expression level in the rats at P14 was significantly lower than that at P35. Multiple regression analysis revealed that the expression levels of RBP4 in rats that developed maternal hypothyroidism during pregnancy were significantly correlated with the levels of TSH secretion. Therefore, RBP4 serves as an important factor during the prediction of early renal damage in rats that develop maternal hypothyroidism during pregnancy. SIRT3 expression in rats with maternal hypothyroidism during pregnancy at P35 was significantly higher than that in normal controls. However, SIRT3 expression in rats with maternal hypothyroidism during pregnancy at P35 was lower than that at P14. We assume that at the early damage stage, the renal mitochondria in the rats with maternal hypothyroidism during pregnancy were occluded and the expression level of RBP4 was increased (30). Long-chain SIRT3 found in the body removes N-end mitochondria, which is the targeting sequence in matrix processing peptidase hydrolysis (31). Long-chain SIRT3 is cut into short-chain SIRT3 that can enter the mitochondria and inhibit CypD activity through mitochondrial membrane channel transition pore opening and deacetylation (32). Therefore, activated mitochondrial autophagy can remove damaged mitochondria, promote mitochondrial biosynthesis, and maintain the normal functioning of cells (33). With the progression of the disease, the expression level of RBP4 increased. Because traumatic stimulation exceeds the mitochondrial quality regulation limit, mPTP opening is not inhibited. However, the mitochondrial autophagy pathway exists in the decompensated state, which leads to an increase in the apoptotic factor, cell death, and kidney tissue damage.

## CONCLUSION

In summary, our findings demonstrate that the activation of the RBP4/PiC/SIRT3 signaling pathway could be involved in the regulation of the opening/closing of the renal mPTP in rats that develop maternal hypothyroidism during pregnancy. Such findings may provide a theoretical basis for thyroid development in fetuses.

## AUTHOR CONTRIBUTIONS

Ren F, Huang R, Li L and Chen D contributed in bioinformatics analysis and writing of the manuscript. Gan H, Deng H and Wang H contributed in discussion of the study findings. Chen D contributed in discussion and comments on an earlier version of the manuscript. All authors have read and approved the final manuscript.

## Figures and Tables

**Table 1:** Baseline clinical and laboratory characteristics of the study population (xˉ±s).

Groups		Weight (g)	Kidney index	TT4 (µg/dl)	TSH (mIU/L)
OH group (n=20)	P14	28.72±1.08#	9.79±0.89#	2.22±0.14	0.12±0.03
	P35	57.78±3.03# ⋇	11.12±0.64# ⋇	2.02±0.11# ⋇	0.60±0.07# ⋇
SCH group (n=20)	P14	45.82±1.07* ▾	7.98±0.58* ▾	2.30±0.12	0.12±0.02
	P35	128.22±2.49* ▾⋇	9.19±0.38▾ ⋇	2.78±0.13▾ ⋇	0.16±0.02▾ ⋇
CON group (n=20)	P14	56.12±1.60	6.70±0.50	2.38±0.17	0.10±0.02
	P35	130.78±2.95⋇	8.81±0.38⋇	2.91±0.20⋇	0.15±0.01⋇

Values are expressed as the mean±standard deviation or number. Control group (CON); Overall hypothyroidism group (OH); Subclinical hypothyroidism group (SCH)

# *p*<0.05, CON *vs.* OH;

* *p*<0.05, CON *vs.* SCH;

▾ *p*<0.05, OH *vs.* SCH;

⋇ *p*<0.01, P14 *vs.* P35.

**Table 2:** The differences in the degree of mPTP opening and PiC mRNA expression among the groups (xˉ±s).

	mPTP opening degree			PiC mRNA expression		
	OH group (n=20)	SCH group (n=20)	CON group (n=20)	OH group (n=20)	SCH group (n=20)	CON group (n=20)
P14	1.12±0.06#	1.07±0.06*	0.56±0.03	19.07±0.33#	18.59±0.75*	12.44±0.63
P35	2.01±0.10# ⋇	1.43±0.05* ▾ ⋇	0.66±0.08⋇	28.58±0.82# ⋇	21.73±0.46* ▾ ⋇	16.08±0.77⋇

Values are expressed as the mean±standard deviation or number. Control group (CON); Overall hypothyroidism group (OH); Subclinical hypothyroidism group (SCH)

# *p*<0.001, CON *vs.* OH;

* *p*<0.001, CON *vs.* SCH;

▾ *p*<0.001, OH *vs.* SCH;

⋇ *p*<0.01, P14 *vs.* P35.

**Table 3:** Comparison of the expression levels of PiC, SIRT3 and RBP4 among the groups (xˉ±s).

	PiC			SIRT3			RBP4		
	OH group (n=20)	SCH group (n=20)	CON group (n=20)	OH group (n=20)	SCH group (n=20)	CON group (n=20)	OH group (n=20)	SCH group (n=20)	CON group (n=20)
P14	23.10±0.88#	22.32±0.82*	12.22±0.55	10.59±0.57#	12.88±0.69* ▾	14.83±0.66	23.07±0.77#	16.81±1.07* ▾	13.13±0.58
P35	34.46±1.05# ⋇	28.26±0.53* ▾ ⋇	14.85±0.64⋇	6.14±0.56# # ⋇	9.64±0.52* * ▾ ⋇	12.12±0.76⋇	34.57±0.84# ⋇	24.58±0.91* ▾ ⋇	15.92±0.71⋇

Values are expressed as mean±standard deviation or number. CON, Control group; OH, Overall hypothyroidism group; SCH, Subclinical hypothyroidism group.

# *p*<0.001, CON *vs.* OH;

* *p*<0.001, CON *vs.* SCH;

▾ *p*<0.001, OH *vs.* SCH;

⋇ *p*<0.01, P14 *vs.* P35.
